# Supplementary material for: Where is communication breaking down? Narrative tensions in obesity-in-pregnancy clinical encounters
Source: PLoS One. 2025 Feb 10;20(2):e0318514. doi: 10.1371/journal.pone.0318514 (PMC11809800; doi:10.1371/journal.pone.0318514)
Supplement: Attachment — Submitted filename: 2024_Annual_Study Renewal-e6 17-0316-E.pdf [file pone.0318514.s001.pdf]

Sinai Health | Mount Sinai Hospital
Joseph & Wolf Lebovic Health Complex

Research Ethics Board

700 University Avenue, 8th fl., Suite 8-600
Toronto, Ontario, Canada, M5G 1Z5
t: (416) 586-4875 f: (416) 586-4715
www.mtsinai.on.ca

Notification of REB Continued Approval

Date: March 27, 2024

To: Dr. Rohan D'Souza
Department of Obstetrics & Gynaecology
Division of Maternal-Fetal Medicine
McMaster University
699 Concession St.
Hamilton, Ontario
L8V 5C2 Canada

Re: 17-0316-E
Developing a Core-outcome Set for Studies Involving Antepartum or Peripartum Interventions/exposures in Pregnant Obese Women (Body Mass Index>30)

**REB Review Type:**	Delegated
**REB Initial Approval Date:**	26 April, 2018
**REB Expiry Date:**	26 April, 2025

Consent Form(s) Currently Approved for Use:
Consent Form: Appendix A: Consent Focus Group Interviews – Patients (dated: 22-Jun-2018)
Consent Form: Appendix B: Consent Individual Interviews - Healthcare Professionals (dated: 22-Apr-2021)
Consent Form: Appendix I: Consent - Consensus Meeting (dated: 09-Apr-2019)
Consent Form: Appendix L: Consent Delphi Invite Non-Professionals (dated: 23-Apr-2018)
Consent Form: Appendix M: Consent Delphi Invite Professionals (dated: 23-Apr-2018)
Consent Form: Appendix W: Consent One-on-One Interviews – Patients (dated: 22-Apr-2021)

The above-named study has received continued approval from the Mount Sinai Hospital Research Ethics Board until the expiry date noted above. Additionally, the REB has approved the above mentioned consent form. If the study is expected to continue beyond the expiry date, you are responsible for ensuring the study receives re-approval. The REB must also be notified of the completion or termination of this study and a final report provided.

If, during the course of the research, there are any serious adverse events, confidentiality concerns, changes in the approved project, or any new information that must be considered with respect to the project, these should be brought to the immediate attention of the REB. In the event of a privacy breach, you are responsible for reporting the breach to the MSH REB and the MSH Corporate Privacy Office (in accordance with Ontario health privacy legislation – Personal Health Information Protection Act, 2004). Additionally, the MSH REB requires reports of inappropriate/unauthorized use of the information. As the Principal Investigator, you are responsible for the ethical conduct of this study.

The MSH Research Ethics Board operates in compliance with the Tri-Council Policy Statement 2, ICH/GCP Guidelines, Part C, Division 5 of the Food and Drug Regulations of Health Canada, Part 4 of the Natural Health Product Regulations, and Part 3 of the Medical Devices Regulations.

During the COVID-19 Publicly Declared Emergency, the REB continues to review and approve submissions, but initiation or implementation of newly approved submissions will be contingent upon evolving institutional policies and guidelines. Principal Investigators are encouraged to consult with their Department Heads for further guidance.

Sincerely,

Beren Avci, BSc, MA
Research Ethics Coordinator, Research Ethics Board

For: Vibhuti Shah, MD, FRCPC, MSc
Chair, Mount Sinai Hospital Research Ethics Board
