# Supplementary material for: Where is communication breaking down? Narrative tensions in obesity-in-pregnancy clinical encounters
Source: PLoS One. 2025 Feb 10;20(2):e0318514. doi: 10.1371/journal.pone.0318514 (PMC11809800; doi:10.1371/journal.pone.0318514)
Supplement: Attachment — Submitted filename: Response to Reviewers - Jan 2025.docx [file pone.0318514.s003.docx]

**Response to Reviewers**

Dear editor and reviewers,

Thank you kindly for returning this manuscript, and for your feedback. I have outlined our responses and any changes below. The responses are initialled by Rachel Dadouch (RD) – see Pages 1 and 2. The clean and marked versions of the manuscript will be attached as well.

Thank you once again,

Rachel, on behalf of the authors of *Where is communication breaking down? Narrative tensions in obesity-in-pregnancy clinical encounters*

Journal Requirements:

Please review your reference list to ensure that it is complete and correct. If you have cited papers that have been retracted, please include the rationale for doing so in the manuscript text, or remove these references and replace them with relevant current references. Any changes to the reference list should be mentioned in the rebuttal letter that accompanies your revised manuscript. If you need to cite a retracted article, indicate the article’s retracted status in the References list and also include a citation and full reference for the retraction notice.

[Note: HTML markup is below. Please do not edit.]

Reviewers' comments:

Reviewer's Responses to Questions

**RD**: We have reviewed the reference list and made edits to entries (e.g. to links) that we noticed may be incorrect. We do not see any other incorrect reference entries beyond these changes. Kindly let us know if we have missed any errors. We did not remove citations throughout the last iteration; we arranged their order in our manuscript text.

**Comments to the Author**

1. If the authors have adequately addressed your comments raised in a previous round of review and you feel that this manuscript is now acceptable for publication, you may indicate that here to bypass the “Comments to the Author” section, enter your conflict of interest statement in the “Confidential to Editor” section, and submit your "Accept" recommendation.

Reviewer #1: All comments have been addressed

Reviewer #2: (No Response)

2. Is the manuscript technically sound, and do the data support the conclusions?

The manuscript must describe a technically sound piece of scientific research with data that supports the conclusions. Experiments must have been conducted rigorously, with appropriate controls, replication, and sample sizes. The conclusions must be drawn appropriately based on the data presented.

Reviewer #1: Yes

Reviewer #2: Yes

3. Has the statistical analysis been performed appropriately and rigorously?

Reviewer #1: Yes

Reviewer #2: Yes

4. Have the authors made all data underlying the findings in their manuscript fully available?

The [PLOS Data policy](http://www.plosone.org/static/policies.action#sharing) requires authors to make all data underlying the findings described in their manuscript fully available without restriction, with rare exception (please refer to the Data Availability Statement in the manuscript PDF file). The data should be provided as part of the manuscript or its supporting information, or deposited to a public repository. For example, in addition to summary statistics, the data points behind means, medians and variance measures should be available. If there are restrictions on publicly sharing data—e.g. participant privacy or use of data from a third party—those must be specified.

Reviewer #1: Yes

Reviewer #2: Yes

5. Is the manuscript presented in an intelligible fashion and written in standard English?

PLOS ONE does not copyedit accepted manuscripts, so the language in submitted articles must be clear, correct, and unambiguous. Any typographical or grammatical errors should be corrected at revision, so please note any specific errors here.

Reviewer #1: Yes

Reviewer #2: Yes

6. Review Comments to the Author

Please use the space provided to explain your answers to the questions above. You may also include additional comments for the author, including concerns about dual publication, research ethics, or publication ethics. (Please upload your review as an attachment if it exceeds 20,000 characters)

Reviewer #1: Thank you for addressing my concerns and completed the necessary edits. The methodology is now clearer and it explains the authors' intention for the study and how it is conducted to achieve the outcomes observed.

Reviewer #2: Please include the BMI data in Table 1. This will give readers a better sense of the severity of the obese group recruited

**RD**: Due to the nature of this study, we are hesitant to include the BMI data, as noted before on account of stigmatizing based on BMI values. This also relates to the limitations of BMI as a diagnostic tool for obesity, specifically with this study. For example, one may presume that a higher BMI is associated to some of these stories and findings, however the BMI value does not depict the nature of the obesity and the issues that may arise. Someone with a BMI of 31 may have abdominal adiposity that translates to the challenges during ultrasound appointments alluded to in this manuscript, whereas another patient with a BMI of 40 with their adiposity distributed elsewhere (and not abdominally) may not have these stories relevant to them. Including the BMI data for this narrative story can lead to readers falsely making conclusions about BMI categories. The stories and narrative tensions in this manuscript can apply to any individual with obesity, despite the BMI value. Kindly let us know if we can maintain our decision to omit the data on BMIs for the patient participants. To address this comment, we will however incorporate this consideration in our future directions. Please refer to Line 763, where we note that there is room in this research area to explore the relationship between BMI categories and the relevance of certain narrative tensions or stories: “Lastly, there is potential in this research area to explore the relationship between these narrative tensions with the different obesity categories and patient health characteristics (including BMI values). Differences in adiposity distribution and BMI categories may relate to certain patient experiences, and accordingly can be further explored.”

7. PLOS authors have the option to publish the peer review history of their article ([what does this mean?](https://journals.plos.org/plosone/s/editorial-and-peer-review-process#loc-peer-review-history)). If published, this will include your full peer review and any attached files.


If you choose “no”, your identity will remain anonymous but your review may still be made public.


**Do you want your identity to be public for this peer review?** For information about this choice, including consent withdrawal, please see our [Privacy Policy](https://www.plos.org/privacy-policy).

Reviewer #1: No

Reviewer #2: No
